# Interoperability of telemonitoring data in digital health solutions: a scoping review

**DOI:** 10.3389/fdgth.2025.1502260

**Published:** 2025-04-22

**Authors:** Diogo Martins, Simon Lewerenz, Anderson Carmo, Henrique Martins

**Affiliations:** ^1^ ISCTE Business School, University Institute of Lisbon, Lisbon, Portugal; ^2^ Faculdade de Ciências da Saúde, Universidade da Beira Interior, Covilhã, Portugal

**Keywords:** eHealth, cross-border exchange of health data, interoperability, telemonitoring, standards, technical standard, cross-border healthcare

## Abstract

**Objectives:**

This scoping review explores the existing literature on the interoperability of telemonitoring systems in cross-border healthcare settings. It focuses on identifying technical standards, enablers, and barriers to effective telemonitoring data exchange across healthcare systems and geographies.

**Methods:**

A systematic search was conducted across databases (MEDLINE, PubMed, ISI Web of Knowledge, DBLP, and Scopus) from January 2000 to May 2023, using keywords such as “telemonitoring”, “interoperability”, “technical standards”, and “cross-border data exchange”. Eligibility criteria included peer-reviewed studies examining the interoperability of telemonitoring systems across healthcare providers and cross-border settings. A total of 861 studies were identified, and 25 met the inclusion criteria.

**Results:**

The review identified diverse technical standards, including HL7 FHIR, ISO/IEEE 11073, and IHE profiles, used in telemonitoring systems. However, significant gaps were found in the literature regarding the operational challenges of telemonitoring systems, particularly in cross-border contexts. Many studies focused on technical aspects, with fewer addressing organizational and legal issues. Data transport types, such as Bluetooth and REST APIs, were mentioned, but no common standard for data exchange between devices was identified.

**Discussion:**

The findings highlight the need for further research on the deployment of telemonitoring systems, particularly in cross-border contexts. The lack of harmonization in technical standards poses a barrier to achieving seamless interoperability. The review calls for the development of a robust framework to support telemonitoring integration across healthcare systems.

**Conclusions:**

While telemonitoring shows promise in improving healthcare delivery, significant interoperability challenges remain. Developing common standards at the European level is essential to enhance cross-border telemonitoring services and patient care.

## Introduction

Telemonitoring, which involves the remote monitoring of patients’ health data via digital devices, has become an integral part of modern healthcare systems, revolutionizing the way patient care is delivered and managed (1–3). Telemonitoring enables continuous, real-time tracking of patients’ physiological parameters. It offers numerous benefits. These include cost-effective delivery of healthcare, reduced need for face-to-face consultations and clinic visits, enhanced quality of care, and improved patient self-management and compliance (4–8). This approach not only alleviates the burden on healthcare systems, but also empowers patients to take an active role in managing their health.

Telemonitoring solutions have become particularly important due to several critical factors. The rapid expansion of the aging population necessitates ongoing healthcare support and regular health assessments (9). Concurrently, the increasing prevalence of chronic diseases requires continuous monitoring and management for affected individuals (10). Furthermore, the rise in healthcare costs emphasizes the need for cost-effective solutions that can reduce the frequency of in-person consultations and hospital admissions (9). Telemonitoring addresses these needs by providing real-time health data, and facilitating timely interventions, thus improving overall patient outcomes.

One significant issue with the effective implementation and benefits of telemonitoring is the challenge posed by interoperability and cross-border exchange. This is particularly relevant for Europe considering the free movement of individuals within the European Union. All EU citizens and their family members have the right to move and reside freely within the EU. This is a fundamental right established by Article 21 of the Treaty on the Functioning of the European Union and Article 45 of the EU Charter of Fundamental Rights (11, 12).

However, addressing interoperability and cross-border exchange is not only critical for supporting the mobility of individuals but also for improving healthcare outcomes globally, as many countries face similar challenges in ensuring seamless healthcare data exchange. Ensuring that telemonitoring systems can seamlessly share and interpret data across different healthcare providers and countries is essential to maintain continuity of care and enhance healthcare delivery (13, 14).

According to the Healthcare Information and Management Systems Society (HIMSS), interoperability is defined as the capability of various information systems, devices, and applications to access, exchange, integrate, and collaboratively utilize data efficiently. This has to occur in a coordinated manner both within and across organizational, regional, and national boundaries. The goal is to ensure the timely and seamless transfer of information, ultimately optimizing the health outcomes for individuals and populations worldwide (15). Interoperability issues can significantly limit or compromise the seamless exchange of health data between different telemonitoring systems, healthcare providers, and countries. Although achieving interoperability is technically feasible for most providers, various technological, organizational, and environmental factors can impede its implementation (16). This is due to interoperability being a matter affecting a wide range of stakeholders including the public, health professionals, and the private sector (17). Addressing these challenges is crucial to ensure the effective integration of telemonitoring solutions across diverse healthcare settings. This allows to optimizing patient care and enhancing healthcare delivery on a broader scale. Indeed, in a recent review, a key regulatory consideration and policy implication highlighted was the development of standardized telemedicine practices on a global scale to facilitate cross-broader telehealth (18).

Importantly, the use of technical standards is essential to ensure that diverse devices and applications used for telemonitoring can communicate seamlessly (19) across different health systems in the EU, as previously highlighted by the European Commission in a call for the harmonization of medical device standards (20). Without standardized protocols, health data captured by telemonitoring devices may not be consistently readable or usable when shared across borders, impeding the continuity of care. Standards for device-to-device interoperability are essential here (19), as they ensure that data generated by various telemonitoring devices can be effectively communicated and processed.

The eHealth ecosystem at European level aims to facilitate safe and efficient cross-border exchange of data for healthcare and research purposes, proposing the European Electronic Health Record Exchange format (EEHRxF) as an EU-wide standard (21). The HL7 FHIR standard is one of the standards recommended by the eHealth Network for this purpose. (22) The European Health Data Space (EHDS) and the European Electronic Health Record Exchange Format (EEHRxF) aim to address these needs by establishing frameworks that facilitate both the direct communication between devices and the standardized structuring of this information within EHRs (21, 22). These initiatives are crucial in overcoming the technical and semantic challenges that currently limit cross-border telemedicine, paving the way for a more cohesive and reliable healthcare experience for patients and providers across the EU. By elaborating on these points, we emphasize that standards are not merely a technical requirement but a foundational need for secure, efficient, and continuous healthcare across the EU.

The principal aim of this scoping review is to map the existing literature on the interoperability of cross-border telemonitoring services with a focus on technical standards and interoperability issues. Taking such action had previously been called for (23). This review seeks to provide a comprehensive understanding of the current landscape. It aims to identify and analyse the enablers and barriers to effective data exchange and integration across different healthcare systems and geographical boundaries. This knowledge will support the development of strategies to overcome interoperability challenges, ensuring seamless, efficient, and patient-centred telemonitoring solutions. Ultimately, the insights gained from this review will inform future research and policy efforts aimed at optimizing telemonitoring services to enhance healthcare delivery and patient outcomes globally.

The main research questions were: (1) What evidence of using interoperable telemonitoring solutions exist and what is its impact on patient care? (2) Which standards or communication protocols between different telemonitoring solutions? (3) Which interoperability frameworks current exists that supports the deploy or understanding on a multi-dimensional level, the implementation of telemonitoring solutions? Thus, our specific objective was to summarize the results of previous studies that focused on technical artifacts or specifications, architecture, standards or guidelines for telemonitoring solutions**.**

## Methods

This scoping review was performed according to Preferred Reporting Items for Systematic Reviews and Meta-Analyses extension for Scoping Reviews (PRISMA-ScR) statement (24).

### Search strategy

A literature search was conducted from January 2000 to May 2023 in the following databases: MEDLINE, PubMed, ISI Web of Knowledge, DBLP and Scopus. The following keywords and Medical Subject Headings (MeSH) terms were used:

• Technical standards OR Architecture AND telemonitoring;
• Telemonitoring AND Technical standards OR Telemonitoring AND Architecture;
• Telemonitoring AND standards AND Interoperability;
• Telemonitoring AND FHIR AND Interoperability;
• Telemonitoring AND FHIR AND Interoperability OR telemonitoring data sharing architecture;
• Telemonitoring AND cross country.

### Eligibility criteria

Inclusion criteria were full-text research articles written in all languages, with English and Portuguese articles read by the author. Articles in other languages were translated as needed. The articles had to be published in peer-reviewed journals and assess telemonitoring solutions, specifically in cross-border settings and cross-organizations. They needed to focus on the benefits, use, and impact of interoperable telemonitoring solutions. They should have different providers or systems, examine technical artifacts or specifications, IT architectures, technical standards or guidelines for telemonitoring solutions, and evaluate the impact and benefits of using telemonitoring solutions based on a common technical framework or international standards. The studies also had to address the enablers and barriers of using telemonitoring solutions in continuous monitoring, non-continuous monitoring, and home care monitoring.

Studies were excluded if they (1) assessed only the benefits of EHRs in general (i.e., did not focus on mobile health records), or (2) assessed the use of telemonitoring solutions in areas not related to healthcare.

### Study selection

Articles were identified through database searches. To identify additional publications, reference lists of identified studies and relevant reviews were manually checked. After duplicates were removed, titles and abstracts resulting from application of the first author search strategy were reviewed for eligibility by a single reviewer according to the eligibility criteria. Uncertainty and doubts were discussed with a third researcher, and a consensus was reached. Potentially relevant studies were identified, and the full texts were obtained. These studies were reviewed and selected for final inclusion according to inclusion and exclusion criteria for full-text review by the first author.

**Figure 1:**
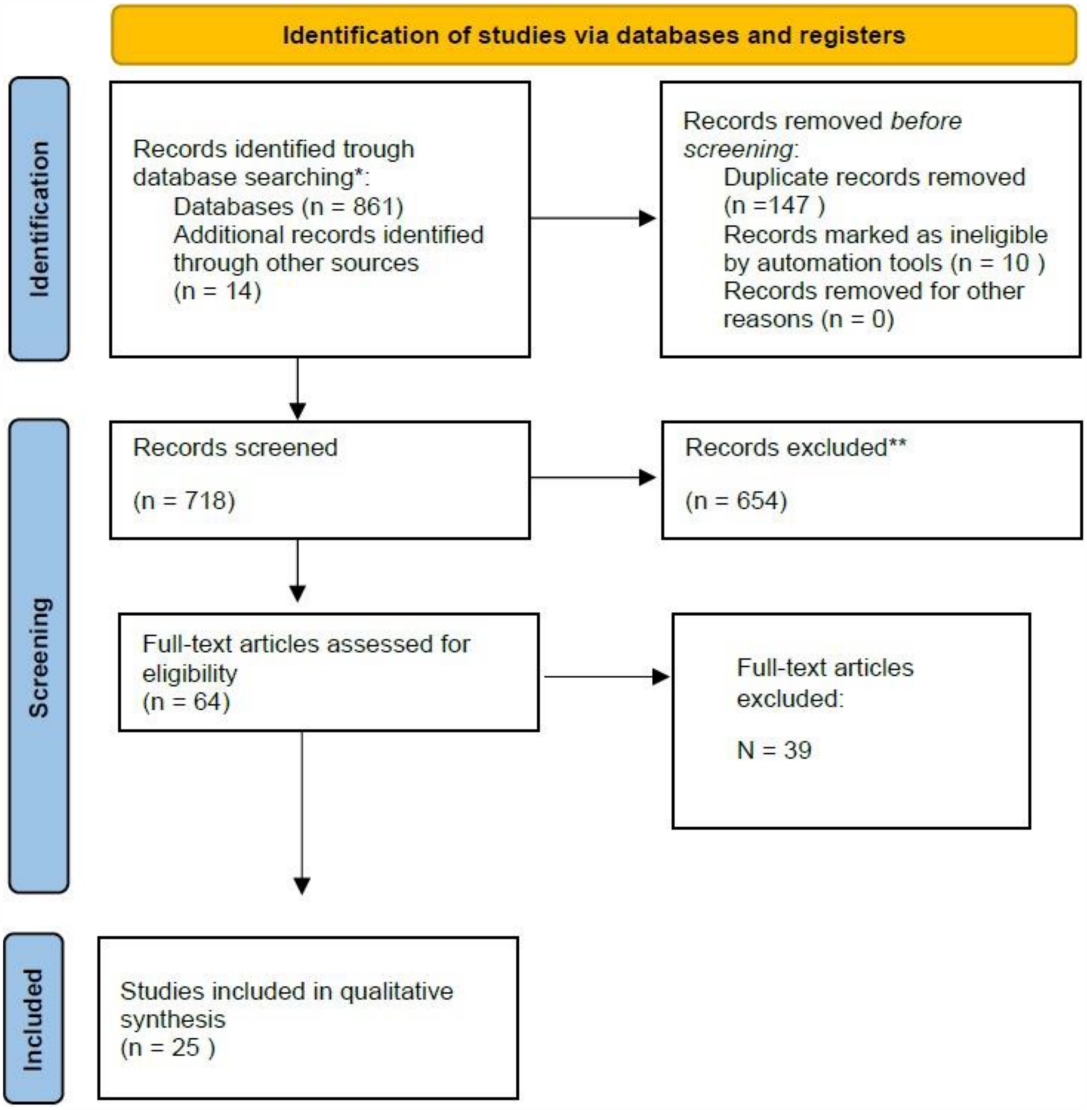
Flowchart of study selection.

A total of 861 studies were identified from all databases and search methods. After full text-review a total of 25 studies met all criteria and were considered for this review (see Figure 1; Table 1).

**Table 1:** Reasons of the exclusion criteria.

Reasons of exclusion	Number of articles that did not meet each criterion
Use of telemonitoring solutions, devices, or remote monitoring technologies aimed at collecting, transmitting, and analyzing patient health data from a distance to facilitate clinical decision-making and continuous patient care (Intervention/Exposure)	19
Studies focusing on telemonitoring and interoperability or telemonitoring data-sharing architecture, including frameworks, protocols, and systems designed to enable seamless exchange of telemonitoring data across different platforms, organizations, and borders. (Comparator)	24
Research assessing telemonitoring solutions specifically in cross-border settings or across multiple healthcare organizations, highlighting challenges and solutions related to data exchange, system integration, and care coordination. (Comparator)	32
Studies examining technical artifacts such as software components, technical specifications, IT architectures, communication protocols, and technical standards or guidelines that ensure the interoperability of telemonitoring solutions across diverse healthcare systems. (Comparator)	27
Research assessing the benefits, use, and impact of telemonitoring solutions that achieve interoperability with different providers, organizations, or systems, particularly in diverse geographical or organizational contexts. (Outcome)	25
Studies focusing on technical artifacts, specifications, IT architectures, and the application of technical standards or guidelines for telemonitoring solutions that ensure secure, reliable, and standardized data exchange. (Outcome)	25
Evaluations of the impact and benefits of telemonitoring solutions that operate within a common technical framework or adopt international standards to enhance data sharing, patient outcomes, and system efficiency. (Outcome)	25
Policy makers involved in healthcare regulation and digital health strategies; healthcare professionals utilizing telemonitoring for patient care; patients using telemonitoring devices and platforms; citizens impacted by digital health initiatives; and standards development organizations (SDOs) responsible for creating and maintaining technical standards for healthcare data interoperability. (Population)	18
Full text not available	2
Full text not accessible	4
Full text duplicate	1

Articles were excluded based on multiple criteria.

In Table 1, we present the main reasons for the exclusion of the 39 articles. It is important to note that some articles were excluded based on multiple criteria.

### Data extraction and synthesis

**Figure 2:**
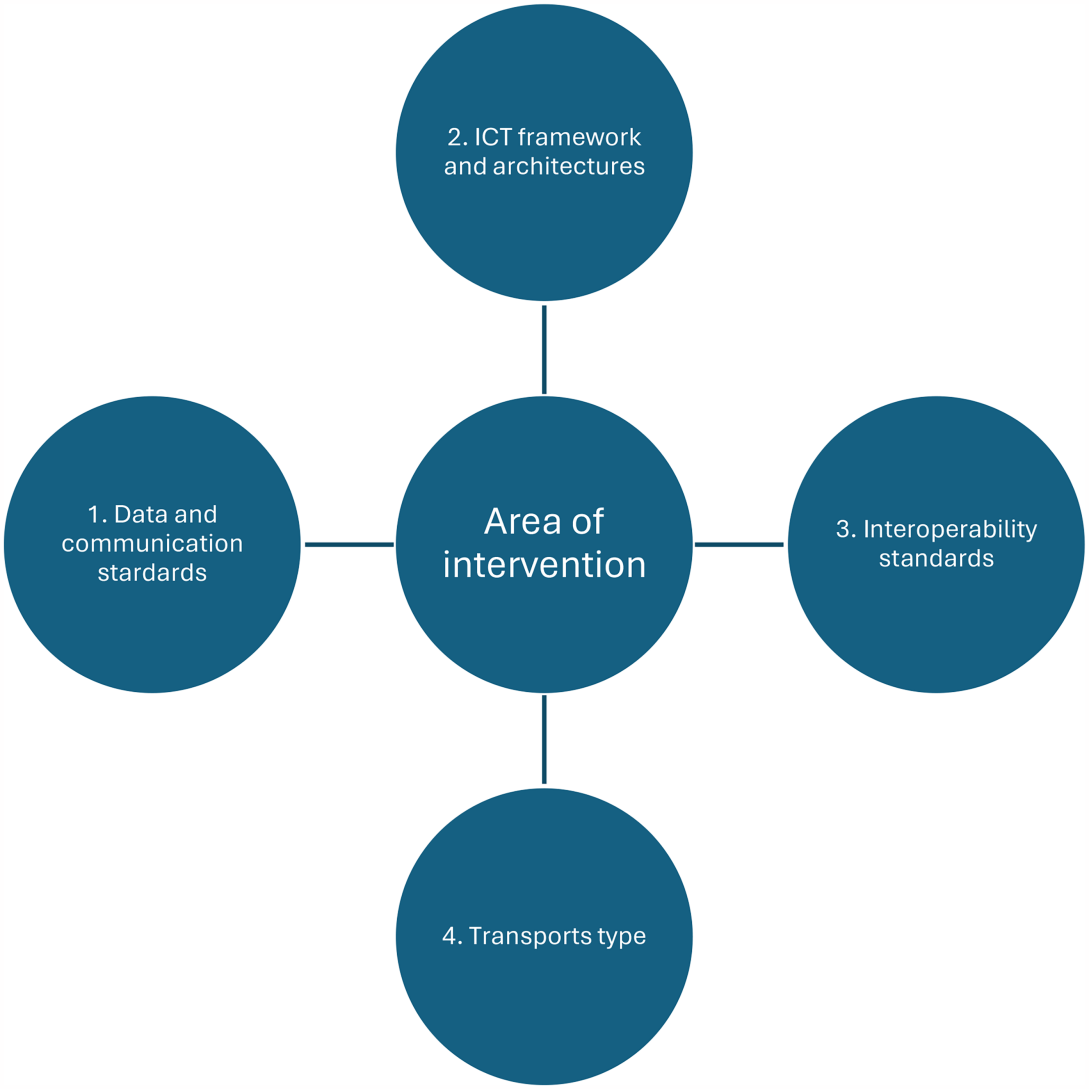
Technical domain for narrative synthesis.

Relevant data was extracted from each included article on the following parameters: country of origin, year, and type of study. Also, a narrative synthesis was conducted, organized according to technical domain (see Figure 2).

## Results

### Study characteristics

The studies included were published between 2005 and 2022, with an increasing number of publications since 2014. Most studies were conducted in Italy (*n* = 5; 20%) and Spain (*n* = 5; 20%), but there was a wide variety of other locations (Austria, Belgium, China, France, Germany, Romania, Panama, and the United Kingdom).

In terms of study methodologies, the majority were observational (*n* = 23, 92%), with 13 focusing on conceptual development (52%), 6 on design and implementation (24%), 4 on evaluation and assessment (16%), and 2 on reviews and analyses (8%). Most of the articles did not specify the sample (64%), whilst 9 studies were conducted with specific patient groups (e.g., focusing on chronic diseases, cardiac diseases, COPD, and Parkinson's, mostly in elderly populations).

The main focus was on technical aspects of interoperability for telemonitoring solutions according to the Refined eHealth European Interoperability Framework (ReEIF) (25). With 25 of the studies reporting on technical aspects, the included papers also recounted information related to Legal aspects (2 studies), organizational aspects (16 studies), semantic aspects (9 studies). Detailed article characteristics can be found as Supplementary Material.

### Technical areas of intervention

To analyse the results, we organised them into four technical areas: Data and communication standards, ICT framework and architectures, Interoperability standards, Transport types. This categorisation was inspired by the “Defining an Open Platform” documentation from Apperta Foundation^1^ and the OSI Model^2^. While these technical areas are mapped to their primary OSI layers in Table 2. We acknowledge that many standards span multiple layers rather than being confined strictly to a single OSI level. Our categorisation reflects the primary impact areas of each technical area, while allowing for flexibility across multiple layers depending on the specific role and application of each standard.

**Table 2:** Technical areas relation to OSI model.

Area	Definition	OSI layers
Data and communication standards	These standards dictate the formats, rules, and protocols for data exchange and communication between systems	Primarily operate at the Application, Presentation, and Session layers
ICT framework and architectures	These are comprehensive guidelines and structures for designing and managing IT infrastructure, including policies, practices, and tools.	multiple layers but often emphasize the higher layers (Application, Presentation, Session) for ensuring overall system design integrity and interoperability
Interoperability standards	Standards that ensure different systems, devices, and applications can work together seamlessly	Cross several layers but focus heavily on the Application, Presentation, and Session layers to enable smooth data exchange and functional integration
Transport types:	Methods and protocols used to transfer data between systems over a network	Operate at the Transport layer, ensuring reliable data transmission and error handling

An overview of the analysis is provided with further details as Supplementary Material.

### Data and communication standards

Various standards used in telemonitoring solutions were identified. HL7 standards, including HL7 FHIR ^3^and CDA^4^, were referenced in 8 studies. ISO/IEEE 11073 ^5^standards for device communication appeared in 9 studies. REST API or REST APIs FHIR based were noted in 4 studies, while XML^6^ was referenced in 5 studies. IT infrastructure from IHE^7^ was mentioned in 1 study. Additionally, 3 studies referred to other standards without a specific standard.

### ICT framework and architectures

The analysis identified diverse standards and frameworks used in telemonitoring solutions. HL7 standards, including FHIR and CDA, were referenced in 8 studies, while ISO/IEEE 11073 standards appeared in 9 studies. REST APIs, particularly FHIR-based, were mentioned in 4 studies, with XML referenced in 5 studies. IT infrastructure from IHE was noted in 1 study, with the IHE Profile Framework specifically referred to in 6 studies. The Continua Alliance framework was mentioned in 1 study, conceptual frameworks with RESTful services in 4 studies, and SOA or XML frameworks in 4 studies. Additionally, 3 studies referred to other standards without a specific partner, and 11 studies mentioned other frameworks without specific standardization.

### Interoperability standards

The analysis identified several studies focusing on various IT infrastructure and interoperability standards used in telemonitoring solutions. Studies referring to IT infrastructure from IHE, including HL7 standards, totalled 10. ISO/IEEE 11073/IEEE 1073 standards were referenced in 11 studies. RestAPI or REST APIs FHIR-based methods were mentioned in 2 studies, while the Continua Framework was noted in 1 study. Additionally, 6 studies utilized other interoperability methods that were non-standard.

### Transport types

In terms transport types used, the analysis identified various transport types used in telemonitoring solutions. Bluetooth, RFID, and ZigBee were referenced in 10 studies. RestAPI or REST APIs FHIR-based methods, along with JSON in FHIR resources, were mentioned in 3 studies. SOP, XML, and similar standards appeared in 4 studies, while 8 studies did not specify the transport types used.

## Discussion

The results of this review revealed a scarcity of technical articles, indicating a significant gap in the literature regarding the detailed technical implementation and operational challenges of telemonitoring systems. This rejoins previous findings (26). Specifically, there is a notable lack of in-depth studies on the deployment and integration of telemonitoring solutions, device-to-device communication protocols, and the practical aspects of achieving seamless interoperability. This gap underscores the need for more comprehensive research focused on the technical aspects, including the configuration of device communication standards like ISO/IEEE 11073 and the integration of these devices with healthcare IT infrastructures using standards such as HL7 FHIR or defined technical workflow based on IHE profiles. Enhanced technical documentation and research can facilitate better understanding, replication, and improvement of telemonitoring solutions, particularly in a cross-border context.

The review indicated some alignment in the use of ISO/IEEE 11073 standards, highlighting their importance in promoting interoperability between devices in telemonitoring systems. These standards are crucial as they ensure that devices from various manufacturers can communicate effectively with each other and with healthcare providers’ EHR systems, enabling value-based care (27). The ISO/IEEE 11073 standards include protocols for real-time data exchange, device plug-and-play interoperability, and standardized data formats, which are essential for seamless integration (26). However, while these standards enable effective local data transmission, their scope is primarily to near-field device communication and does not directly address the broader requirements of cross-border interoperability.

This interoperability is vital for cross-border healthcare, where devices and systems from different regions need to work together without compatibility issues. By adopting ISO/IEEE 11073 standards, healthcare providers can ensure reliable and efficient telemonitoring, ultimately enhancing patient care across different countries. The use of such standard as a common standard for devices interoperability or communication seems and early adopter to be tested and upscaled as generic standards, but as well on cross-border context. While there is some indication of the use of IT infrastructure from the Integrating the Healthcare Enterprise (IHE), including HL7 standards, for telemonitoring purposes, the evidence remains limited and suggests that adoption is still in its early stages. Several studies have shown that these infrastructures are adapted for specific telemonitoring needs (29–33). For instance, HL7 FHIR is used for exchanging healthcare information electronically, providing a robust framework for integrating telemonitoring data with EHR systems. However, the adaptation of these standards often involves customizing profiles to fit specific telemonitoring requirements, such as remote patient monitoring and chronic disease management (28). This adaptability highlights the flexibility of these standards but also emphasizes the need for standardized profiles that cater to telemonitoring in cross-border scenarios.

Despite some popularity for certain franchises, the review identified a lack of common standards for data exchange between devices and EHR systems. Bridging this gap is essential to ensure that data is accessible to patients, researchers, and healthcare providers using a common standard or method. Enhancing interoperability at this level will improve data accessibility and utility, facilitating better patient care and more effective research. The implementation rules and guidelines to be used as baseline for any implementation at European Level, both for cross-border exchange and for country-level report sharing could be based on HL7 FHIR standard, as ongoing harmonization efforts already adopt it for this purpose (22). As the EEHRxF is proposed as an EU standard for healthcare, further exploration by research appears essential to validate or invalidate this.

The review revealed a lack of evidence regarding the use of standards in a cross-border context, with only three studies reflecting on data exchange between different entities. Although these standards theoretically apply to cross-border scenarios, additional aspects defined by the ReEIF need to be addressed (25). Furthermore, 15 studies identified the need to exchange data between different applications and IT providers, highlighting the necessity for further research and development in cross-border interoperability.

Both findings of a variety of different approaches to interoperability and of scarce evidence of interoperability point to the same suggestion: there is the need for a more robust framework around telemonitoring (34). In Europe, the European Health Data Space regulation was adopted in April 2024 and serves as a flagship effort for empowering eHealth (35). In forward-looking countries, telemonitoring is already being included in efforts to implement EHDS requirements (36). Telemedicine is mentioned in different provisions (Recitals 21/22, Article 13), and appears to be one of the next domains to be included in the EHDS (37). Including telemonitoring as one facet of telemedicine in the EHDS through additional implementation acts can help bridge the gaps identified in this research, namely the lack of harmonization and evidence of telemonitoring interoperability services.

It is important to recognize that although the technical aspects of telemonitoring interoperability are fundamental to ensuring seamless data exchange across systems, which is essential for effective telemonitoring in cross-border settings, legal frameworks provide the necessary safeguards for data privacy, security, and patient rights, while organizational structures ensure that policies are effectively implemented and that stakeholders are aligned. Future research should aim to explore these legal and organizational dimensions in greater depth, as addressing these challenges is crucial for achieving comprehensive and equitable healthcare integration across borders.

Overall, the results of this review should be interpreted with caution. One limitation was the use of a single examiner for the preliminary evaluation, which may introduce potential bias; however, other reviewers were consulted in cases of doubt or discussion to ensure balanced and objective assessments. Future studies could benefit from employing a dual-reviewer approach to further enhance the reliability of the evaluation process.

## Conclusions

This scoping review highlighted the diverse range of standards and frameworks utilized in telemonitoring solutions, ranging from ISO/IEEE 11073 to HL7 FHIR standards and IHE profiles, emphasizing the varying approaches to achieving interoperability and effective data communication in healthcare. Additionally, this review uncovered the lack of evidence of use of standards, thus the need for further research to understand how telemonitoring solutions can leverage new healthcare standards to enhance interoperability and accelerate the transition from conceptual studies to practical applications.

Thus, it is essential for healthcare practitioners and policy makers to adopt and promote standardized frameworks for data exchange. Implementing recognized interoperability standards, such as HL7, IHE profiles, and ISO/IEEE protocols, can facilitate secure, efficient, and scalable telemonitoring systems. Policy makers should support initiatives that establish legal and regulatory frameworks for cross-border data sharing, while healthcare organizations should invest in interoperable technologies and staff training. Working towards a robust framework is needed to support practice and research. Internationally funded efforts such as X-eHealth, XpanDH, xShare, or Xt-EHR projects are crucial to foster harmonization. Furthermore, including telemonitoring into legislative frameworks could be pivotal for interoperability developments. In this line of work, it is crucial to establish common standards at the European level to enhance the interoperability of telemonitoring solutions. These standards will enable the healthcare domain to effectively leverage telemedicine technologies, accelerating the transition from conceptual studies to practical applications across Europe.

Future research should continue to explore not only technical solutions but also the legal and organizational enablers of interoperability, ensuring that healthcare systems are well-prepared to deliver integrated care across borders. By incorporating these recommendations, healthcare systems can enhance patient care, improve resource utilization, and foster greater collaboration at both national and international levels.

## Footnotes

^1^Accessed at: https://apperta.org/assets/Apperta_Defining_an_Open_Platform.pdf

^2^“Open Systems Interconnection” is a reference model standardizing the functions of a telecommunication or computing system into seven abstraction layers: Application, Presentation, Session, Transport, Network, Data Link, Physical. Accessed at: https://www.iso.org/standard/20269.html

^3^Health Level Seven International Fast Healthcare Interoperability Resources

^4^Clinical Document Architecture

^5^ISO/IEEE 11073: Health Informatics – Medical/health device communication standards

^6^Extensible Markup Language

^7^IHE: Integrating the healthcare Enterprise

## References

[1] Haleem A Javaid M Singh RP Suman R. Telemedicine for healthcare: capabilities, features, barriers, and applications. Sens Int. (2021) 2:2–12. 10.1016/j.sintl.2021.100117 PMC8590973 34806053

[2] Serrano LP Maita KC Avila FR Torres-Guzman RA Garcia JP Eldaly AS Benefits and challenges of remote patient monitoring as perceived by health care practitioners: a systematic review. Perm J. (2023) 27:100–11. 10.7812/TPP/23.022 37735970 PMC10730976

[3] Ricci G Caraffa AM Gibelli F. Telemedicine as a strategic tool to enhance the effectiveness of care processes: technological and regulatory evolution over the past two decades. Healthcare. (2023) 11(5):734. MDPI. 10.3390/healthcare11050734 36900739 PMC10000912

[4] Boyne JJ Vrijhoef HJ Spreeuwenberg M De Weerd G Kragten J Gorgels AP Effects of tailored telemonitoring on heart failure patients’ knowledge, self-care, self-efficacy and adherence: a randomized controlled trial. Eur J Cardiovasc Nurs. (2014) 13:243–52. 10.1177/1474515113487464 23630403

[5] Sul AR Lyu DH Park DA. Effectiveness of telemonitoring versus usual care for chronic obstructive pulmonary disease: a systematic review and meta-analysis. J Telemed Telecare. (2020) 26:189–99. 10.1177/1357633×18811757 30541375 10.1177/1357633X18811757

[6] van der Burg JMM Aziz NA Kaptein MC Breteler MJM Janssen JH van Vliet L Long-term effects of telemonitoring on healthcare usage in patients with heart failure or COPD. Clinical EHealth. (2020) 3:40–8. 10.1016/j.ceh.2020.05.001

[7] Scholte NT Gürgöze MT Aydin D Theuns DA Manintveld OC Ronner E Telemonitoring for heart failure: a meta-analysis. Eur Heart J. (2023) 44(31):2911–26. 10.1093/eurheartj/ehad320 37216272 PMC10424885

[8] Huygens MWJ Voogdt-Pruis HR Wouters M Meurs MM van Lettow B Kleijweg C The uptake and use of telemonitoring in chronic care between 2014 and 2019: nationwide survey among patients and health care professionals in The Netherlands. J Med Internet Res. (2021) 23:e24908. 10.2196/24908 33938808 PMC8129877

[9] Eurostat. Population Structure and Ageing: Eurostat Statistics Explained. European Commission (2024). Available online at: https://ec.europa.eu/eurostat/statistics-explained/index.php?title=Population_structure_and_ageing (Accessed August 9, 2024).

[10] Vandenberghe D Albrecht J. The financial burden of non-communicable diseases in the European union: a systematic review. Eur J Public Health. (2020) 30:833–9. 10.1093/eurpub/ckz073 31220862

[11] Treaty on the Functioning of the European Union. Fundamental Texts on European Private Law. Luxembourg: European Union (2020). 10.5040/9781782258674.0002

[12] The European Parliament the C and the C. Charter of Fundamental Rights of the European Union. Nice: Official Journal of the European Communities (2000). Available online at: https://www.europarl.europa.eu/charter/pdf/text_en.pdf (Accessed August 9, 2024).

[13] Hayes CJ Dawson L McCoy H Hernandez M Andersen J Ali MM Utilization of remote patient monitoring within the United States health care system: a scoping review. Telemed E Health. (2023) 29(3):384–94. 10.1089/tmj.2022.0111 35819861

[14] Walker DM Yeager VA Lawrence J Mcalearney AS. Identifying opportunities to strengthen the public health informatics infrastructure: exploring hospitals’ challenges with data exchange. Milbank Q. (2021) 99(2):393–425. 10.1111/1468-0009.12511 33783863 PMC8241268

[15] Healthcare Information and Management Systems Society (HIMSS). Interoperability in Healthcare. HISSM Europe (2024). Available online at: https://www.himss.org/resources/interoperability-healthcare (Accessed August 9, 2024)

[16] Walker DM Tarver WL Jonnalagadda P Ranbom L Ford EW Rahurkar S. Perspectives on challenges and opportunities for interoperability: findings from key informant interviews with stakeholders in Ohio. JMIR Med Inform. (2023) 11:e43848. 10.2196/43848 36826979 PMC10007006

[17] Lewerenz S Moen A Martins H. Public value and digital health: the example of guiding values in the national digital health strategy of France. Int J Med Inform. (2025) 196:105794. 39862565 10.1016/j.ijmedinf.2025.105794

[18] Adeghe EP Okolo CA Ojeyinka OT. A review of emerging trends in telemedicine: healthcare delivery transformations. Int J Life Sci Res Arch. (2024) 6(1):137–47. 10.14303/irjbcs.2023.42

[19] Gowda V Schulzrinne H Miller BJ. The case for medical device interoperability. JAMA Health Forum. (2022) 3(1):e214313–e214313. American Medical Association. 10.1001/jamahealthforum.2021.4313 36218857

[20] European Commission. MDCG 2021-5 Guidance on Standardisation for Medical Devices. European Commission (2021). Available online at: https://ec.europa.eu/health/sites/default/files/md_sector/docs/md_mdcg_2021_5_en.pdf (Accessed October 27, 2021)

[21] European Commission. Commission Recommendation of 6.2.2019 on a European Electronic Health Record Exchange Format. European Commission (2019). Available online at: https://digital-strategy.ec.europa.eu/en/library/recommendation-european-electronic-health-record-exchange-format

[22] eHealth Network. eHealth Network Guideline on the Electronic Exchange of Health Data Under Cross-Border Directive 2011/24/EU: Laboratory Results. eHealth Network (2023). Available online at: https://health.ec.europa.eu/document/download/4e8d82ba-87ca-4123-a8d8-a5d895ae8ecc_en?filename=ehealth_ehn-lab-results-guideline_en.pdf (Accessed August 9, 2024)

[23] Lewerenz S Martins D Martins H. Assessing cross-border telemedicine data exchange in the European union: a call to action. Telemedicine E Health. (2024) 30(11):2759–62. 10.1089/tmj.2024.0338 39137057

[24] Tricco AC Lillie E Zarin W O'Brien KK Colquhoun H Levac D PRISMA extension for scoping reviews (PRISMA-ScR): checklist and explanation. Ann Intern Med. (2018) 169(7):467–73. 10.7326/M18-0850 30178033

[25] eHealth Network. Refined EHealth European Interoperability Framework. European Commission (2024). Available online at: https://health.ec.europa.eu/document/download/84839361-c768-4ad5-b7ca-e46b2123fef2_en (Accessed 9, August 2024)

[26] Dias A Martins AI Queirós A Rocha NP. Interoperability in pervasive health: a systematic review. In: Cliquet A Jr Wiebe S Anderson P Saggio G Zwiggelaar R Gamboa H Biomedical Engineering Systems and Technologies. BIOSTEC 2018. Communications in Computer and Information Science. Vol. 1024. Cham: Springer. (2019). p. 279–97. 10.1007/978-3-030-29196-9_15

[27] Zhang X Saltman R. Impact of electronic health record interoperability on telehealth service outcomes. JMIR Med Inform. (2022) 10(1):e31837. 10.2196/31837 34890347 PMC8790688

[28] Schmitt L Falck T Wartena F Simons D. Novel ISO/IEEE 11073 standards for personal telehealth systems interoperability. In: 2007 Joint Workshop on High Confidence Medical Devices, Software, and Systems and Medical Device Plug-and-Play Interoperability (HCMDSS-MDPnP 2007); Boston, MA, USA. New York, NY: Institute of Electrical and Electronics Engineers (IEEE) (2007). p. 146–8. 10.1109/HCMDSS-MDPnP.2007.9

[29] Lanzola G Polce F Tibollo V Quaglini V Wilk S. Designing a testing environment for the CAPABLE telemonitoring and coaching platform. MELECON 2022—IEEE Mediterranean Electrotechnical Conference, Proceedings (2022). 10.1109/MELECON53508.2022.9843001

[30] Finet P Gibaud B Dameron O Le Bouquin Jeannès R. Interoperable infrastructure and implementation of a health data model for remote monitoring of chronic diseases with comorbidities. IRBM. (2018) 39:151–9. 10.1016/j.irbm.2018.03.003

[31] Clarke M de Folter J Verma V Gokalp H. Interoperable end-to-end remote patient monitoring platform based on IEEE 11073 PHD and ZigBee health care profile. IEEE Trans Biomed Eng. (2018) 65(5):1014–25. 10.1109/TBME.2017.2732501 28796600

[32] Yang M Chronaki CE Lüpkes C Thiel A Plößnig M Hinterbuchner L Guideline-driven telemonitoring and follow-up of cardiovascular implantable electronic devices using IEEE 11073, HL7 and IHE profiles. 2011 Annual International Conference of the IEEE Engineering in Medicine and Biology Society; IEEE (2011). p. 3192–6. 10.1109/IEMBS.2011.6090869 22255018

[33] Feng H Shi B Cao X Hong X Duan X Zhong D. The conceptual modeling of interoperability framework of heart sound monitor in the context of an interoperable end-to-end architecture. Telemed E Health. (2019) 25(9):808–20. 10.1089/tmj.2018.0188 30328780

[34] Julesz M. A perspective on the European health data space. Inform Társadalom. (2023) 23:9. 10.22503/inftars.XXIII.2023.4.1

[35] European Health Data Space Regulation. European Parliament. European Commission (2024). Available online at: https://www.europarl.europa.eu/doceo/document/TA-9-2024-04-24_EN.html#sdocta18 (Accessed July 8, 2024)

[36] Hussein R Sareban M Treff G Niebauer J. Roadmap for aligning cardiovascular digital health in Austria with the European Health Data Space (EHDS) ecosystem. Stud Health Technol Inform. (2023) 309:101–5. 10.3233/SHTI230750 37869816

[37] Lewerenz S Zanini A Martins D. Exploring the Implications of the New EHDS Regulation for Telemedicine and the EEHRxF. XpanDH Blog (2024). Available online at: https://xpandh-project.iscte-iul.pt/exploring-the-implications-of-the-new-ehds-regulation-for-telemedicine-and-the-eehrxf/ (Accessed August 8, 2024)

