# Supplementary material for: Interoperability of telemonitoring data in digital health solutions: a scoping review
Source: Front Digit Health. 2025 Apr 22;7:1502260. doi: 10.3389/fdgth.2025.1502260 (PMC12052697; doi:10.3389/fdgth.2025.1502260)
Supplement: Supplementary file 1 [file Datasheet1.docx]

**Supplementary Material**

**General Characteristics**

| Name | Year | Country | Reference |
| --- | --- | --- | --- |
| Lanzola, G | 2022 | Italy | [13] |
| Calderon-Gomez, H, | 2020 | Spain  Panama (??) | [48] |
| P Finet et al, | 2018 | France | [72] |
| Clarke, M, et al, | 2018 | UK | [74] |
| J Schaaf et al, , | 2013 | Germany | [133] |
| Yang, M, et al, , | 2011 | Multinational | [147] |
| Lasierra, N, et al, | 2010 | No country available | [159] |
| Meir, H, | 2009 | Germany | [176] |
| Sabutsch, S, et al | 2022 | Austria | [315] |
| Giacomo, P,et al | 2005 | Italy | [354] |
| Suciu, G, et al | 2015 | Romania | [419] |
| Vandenberk, T, et al | 2019 | Belgium | [454] |
| Jimenez-Fernandez, S, et al, | 2013 | Spain | [463] |
| Vergari, F, et al, | 2012 | Italy | [772] |
| Gazzarata,R , et al, | 2014 | Italy | [776] |
| Lasierra,N, et al, | 2014 | Spain | [782] |
| Piro,NE, et al, | 2014 | Germany | [819] |
| Galarraga,M, et al, | 2007 | Spain | [824] |
| Martínez,I, et al, | 2008 | Spain | [826] |
| de Toledo, P, et al, | 2006 | Spain | [828] |
| Feng, H, et al, | 2018 | China | [831] |
| Urbauer, P, et al, | 2017 | Austria | [832] |
| Gokalp, H, et al, | 2018 | United Kingdom | [834] |
| Kumpusch, H, et al, | 2010 | Austria | [836] |
| Macis, S, et al, | 2020 | Italy | [837] |

Table S1 - General characteristics of included studies (N = 25).

| Year of Publication |  |
| --- | --- |
| 2005 | 1 |
| 2006 | 1 |
| 2007 | 1 |
| 2008 | 1 |
| 2009 | 1 |
| 2010 | 2 |
| 2011 | 1 |
| 2012 | 1 |
| 2013 | 2 |
| 2014 | 3 |
| 2015 | 1 |
| 2017 | 1 |
| 2018 | 4 |
| 2019 | 1 |
| 2020 | 2 |
| 2022 | 2 |

Table S2 - Years of Publication of included studies (N = 25)

| Number of Studies included for full text review | 25 |  |
| --- | --- | --- |
| Countries of Publication |  |  |
| Austria | 3 | 12% |
| Belgium | 1 | 4% |
| China | 1 | 4% |
| France | 1 | 4% |
| Germany | 3 | 12% |
| Italy | 5 | 20% |
| Multinational | 1 | 4% |
| No country available | 1 | 4% |
| Romania | 1 | 4% |
| Spain | 5 | 20% |
| Spain  Panama (??) | 1 | 4% |
| United Kingdom | 2 | 8% |

Table S3 - Countries of Publication of included studies (N = 25)

**Technical Overview and analysis**

| *Studies Characteristics (N = 25)* | **Technical** | **Technical** | **Technical** | **Technical** |
| --- | --- | --- | --- | --- |
| **Study, country [reference] - ID** | **Area of intervetion 1: Data and Communication Standards used (RESTful web-services, SOAP, etc)** | **Area of intervetion 2: ICT framework and Architectures (Continua, ICT Architecture - IHE XDS-profile, etc)** | **Area of intervetion 3: Interoperability Standards used (ISO/IEEE 11073 Personal Health Device (PHD)** | **Area of intervetion 4: Transports types used (HL7, XML,json, etc)** |
| Lanzola, G.; 2022 Italy [13] | HL7 FHIR | Multi-agent architecture, Decision Support System (DSS) Wrapper | HL7 FHIR as a semantic interoperability standard | XML, JSON in FHIR resources |
| Calderon-Gomez, H.; 2020 Spain, Panama [48] | REST protocol, HTTPS server | Microservices architecture, MariaDB, NoSQL (Cassandra, MongoDB) | N/A | JSON, REST API, TLS for client authentication |
| P. Finet et al., 2018 France [72] | HL7 CDA, HL7 v2.6, ISO/IEEE 11073 standards | Telemedicine system with integrated health data model, HL7, IHE integration profiles | HL7 standards, IHE profiles (DEC, XDS), ISO/IEEE 11073 standards | HTTPS for secure data transfer, HL7 messaging |
| Clarke, M, et al., 2018 UK [74] | IEEE 11073, ZigBee Health Care Profile, XML, JSON | ZigBee mesh network supporting health and telecare domains, integration with home automation and light link protocols | IEEE 11073, IHE PCD-01 (a profile of HL7), Continua Guidelines for interoperability | ZigBee for wireless communication, support for IEEE 802.15.4 link layer, sleepy end-device protocol for ultra-low power operation |
| J. Schaaf et al., 2013, Germany [133] | Encrypted data transfer, mobile internet network | .NET Framework, Windows Server 2008 R2, SQL Server 2008 R2, IIS for hosting web services | HL7 for standardized information exchange in healthcare | Mobile internet network for data transmission |
| Yang, M, et al., 2011, Multinational [147] | HL7v2.x ORU messages, ISO/IEEE 11073 nomenclature | Service-oriented architecture for the communication server, HL7 and ISO/IEEE 11073 for data formatting | ISO/IEEE 11073, HL7, IHE Profiles (IDCO, PIX) | Internet-based services for data transmission and reporting |
| Lasierra, N, et al., 2010 No country available [159] | SOAP / HTTPS in system architecture. | HTPPO (Home Telemonitoring Patient Profile Ontology) for clinical guidelines and management policies. | ISO/IEEE 11073 standards for medical device communication. | Use of SOAP and HTTPS implies XML and other web data formats. |
| Meir, H, 2009 Germany [176] | SOAP used to trans XML data packages | Webservices architecture based on .net libraries | HTTP and TCP protocols | Webservices are used connect the server not the telematic device Micro system data exchange format (msd) |
| Sabutsch, S, et al. 2022 Austria [315] | HL7 CDA standard. | ELGA uses elements from e-Reports for sharing machine-readable data. | The Telehealth Note complies with IEEE 11073 and HL7 CDA standard. | Not explicitly discussed |
| Giacomo, P,et al. 2005 Italy [354] | XML for encoding and exchanging ECG telemonitoring data. | Architecture for clinical information exchange integrating telemonitoring signal into XML document. | XML data-encoding standards in telemonitoring systems. | Use of XML implies data formats for encoding and representing ECG signals and clinical information. |
| Suciu, G, et al. 2015 Romania [419] | RFID, WSN technologies, and M2M communication protocols in IoT applications. | Decentralized cloud architecture for E-Health applications using RTUs. | Not explicitly mentioned | Fog computing's role in IoT and cloud security. |
| Vandenberk, T, et al. 2019 Belgium [454] | The platform utilizes a combination of web-based and mobile technologies, including a smartphone app and cloud-based server architecture for data collection and communication​​. | DHARMA's technical architecture includes a component-based middleware, ensuring flexibility and adaptability for various clinical research needs​​. | Future improvements include integrating with standard information models and communication standards like Health Level 7 and clinical terminologies for interoperability with hospital EMR systems​​. | Not explicitly discussed, but the platform's use of cloud servers and mobile apps implies the use of standard data formats and protocols for transmitting patient data. |
| Jimenez-Fernandez, S, et al., 2013 Spain [463] | Uses wireless sensor networks incorporating standards like ISO IEEE 11073 for environments typical of patient monitoring sensor networks​​ | Innovative user interface not based on TV, PC, or smartphone, employing physical interface, plug-and-play network reconfiguration, and interoperability standards​​ | Challenges and improvements of the ISO IEEE 11073 standard in the context of limited computational power environments​​ | Utilizes IEEE 802.15.4 standard in the 868 MHz band for local communications, enabling low power consumption and incorporating AES hardware encryption​ |
| Vergari, F, et al., 2012 Italy [772] | HL7 v3 CDA R2, XML/RDF structuring. | SOA for information exchange. | HL7 v3 for information exchange. | SSAP based on XML, Bluetooth communication. |
| Gazzarata,R , et al., 2014 Italy [776] | CDA Release 2 (CDA R2) standard. | SOA-based middleware framework. | Healthcare Services Specification Project (HSSP) norms. | Diverse communication technologies integration. |
| Lasierra,N, et al., 2014 Spain [782] | REST WS technology for data communication. | Conceptual framework with RESTful services. | Integration focus for telemonitoring platforms. | HTTPS communication, REST WS secure data exchange. |
| Piro,N.E, et al., 2014 Germany [819] | HL7 v2 and webservices to communicate HL7 to server | User-friendly sensor unit design. | Continua Health Alliance recommendations. ISO/IEEE 11073 Personal Health Device (PHD) | Motion data communication via extended Bluetooth. |
| Galarraga,M., et al., 2007 Spain [824] | ISO/IEEE 11073 standards for telemonitoring. | Continua Alliance framework. | ISO/IEEE 11073 for device standardization. | Bluetooth, Zigbee, USB, RS-232. |
| Martínez,I., et al., 2008 Spain [826] | ISO/IEEE 11073 for personal health devices. | Telemonitoring platform optimization. | Focus on X73-PHD for wearables. | Bluetooth, ZigBee, RFID technologies. |
| de Toledo, P., et al., 2006 Spain [828] | ISO/IEEE 11073/IEEE1073 for personal and wearable devices. | Modular telemonitoring solutions development. | ISO11073/IEEE1073 for sensor/device interoperability. | Bluetooth, ZigBee, RFID for wireless connectivity. |
| Feng, H., et al., 2018 China [831] | ISO/IEEE 11073 standards for heart sound monitors. | End-to-end architecture for heart sound monitoring. ISO/IEEE 11073 Personal Health Device (PHD) compliant model | ISO/IEEE 11073 Personal Health Device (PHD) compliant model | Bluetooth, ZigBee, and RFID in telemonitoring. |
| Urbauer, P., et al., 2017 Austria [832] | ISO/IEEE 11073, Continua Health, HL7 FHIR. | Standards-based architecture for data integration. | RESTful web-services i.e. FHIR based | RESTful web-service, Bluetooth Low Energy and similar wireless tech. |
| Gokalp, H., et al., 2018 United Kingdom [834] | IEEE 11073, IHE PCD-01 of HL7. | IHE PCD-01 of HL7 | IEEE 11073, IHE PCD-01 of HL7 Standards-based sensor and data platform communication. | ZigBee Healthcare Profile, GPRS for sensor data. |
| Kumpusch, H., et al., 2010 Austria [836] | Bluetooth, NFC, HL7 standards. | Not specified | Standardized communication for mobile health integration. | NFC, Bluetooth for device data, UMTS for backend. |
| Macis, S., et al., 2020 Italy [837] | Bluetooth, XML for device compatibility. | SOA for scalable telecare framework. | IHE - ITI Profiles as infrastructured based (Cross-Enterprises Documents Sharing (XDS.b), Patient Identifier Cross-Referencing (PIX), Patient Demographics Query (PDQ) and Document Metadata Subscription (DSUB)) and XML documents to exchange data | Bluetooth for local, HTTPS for cloud data transmission. |

Table S4 - Technical Analysis of the Studies Included
